# A regulatory gap analysis of midwifery to deliver essential reproductive, maternal, newborn, child and adolescent health services in Lao People’s Democratic Republic

**DOI:** 10.1016/j.lanwpc.2023.100960

**Published:** 2023-12-05

**Authors:** Shogo Kubota, Moe Ando, John Murray, Sengmany Khambounheuang, Khampasong Theppanya, Phouvanh Nanthavong, Chankham Tengbriacheu, Malouny Sisavanh, Thongchan Khattiyod, Daisuke Asai, Howard Sobel, Masamine Jimba

**Affiliations:** a WHO Laos, Vientiane, Lao PDR; b WHO Western Pacific Region, Manilla, Philippines; c Department of Health Personnel, Ministry of Health, Vientiane, Lao PDR; d National Mother and Child Health Center, Department of Hygiene and Health Promotion, Ministry of Health, Vientiane, Lao PDR; e Department of Community and Global Health, Graduate School of Medicine, The University of Tokyo, Tokyo, Japan

**Keywords:** Midwifery, Scope of practice, Regulation, Essential health service, Policy gap analysis, Low-middle income country, Maternal and reproductive health, Newborn, Child and adolescent health

## Abstract

**Background:**

In Lao Peoples Democratic Republic, midwives are the main providers of primary reproductive, maternal, newborn, child and adolescent (RMNCAH) services. We analyzed to what extent practice regulations allow midwives to provide nationally defined essential RMNCAH services.

**Methods:**

Stakeholder consultations and document reviews were conducted to identify the essential RMNCAH interventions and care tasks midwives are expected to provide without physicians. These were defined in: 1) the Essential Health Service Package (EHSP) and 2) 18 national standards and guidelines. We then mapped whether midwifery regulations, which provide the legal framework for clinical service provision, supported delivery of these standards to identify regulatory gaps. Data were used to update regulations.

**Findings:**

Midwives were expected to provide 39 RMNCAH interventions without physicians, representing 1100 care tasks. Midwifery practice regulations allowed eight of 39 interventions (20.5%) and 705 of 1100 care tasks (64.1%) at baseline. Of the 31 interventions not allowed for provision by midwives, 83.9% (26) required prescribing and giving medicines, 51.6% (16) ordering and conducting diagnostics, 38.7% (12) making a clinical diagnosis, and 22.6% (7) use of non-pharmacological interventions. The Ministry of Health convened a multi-stakeholder group to revise the midwifery practice regulations, which increased the legally supported interventions and care tasks to 37 (94.9%) and 1081 (98.3%), respectively.

**Interpretation:**

This novel methodology enabled systematic identification and quantification of regulatory gaps in midwifery practice and data-driven revisions. Consequently, regulatory support for delivery of primary RMNCAH interventions vastly improved. The approach can be applied to other clinical cadres, service areas and countries.

**Funding:**

Korea Foundation for International Health Care (KOFIH) supported research operation.

**Research in context**

**Evidence before this study**

Midwives in low- and middle-income countries are often important providers of primary health care. Although the interventions that midwives are expected to provide in primary care settings have been defined, their ability to provide services is often limited by a lack of effective regulatory policy standards. The mismatch between what midwives are legally allowed to provide and what they are required to provide means that essential services are often withheld, limiting access to and quality of care, or provided without legal protection. We wished to determine whether national midwifery regulations in Lao PDR supported the delivery of key reproductive, maternal, newborn, child and adolescent health (RMNCAH) interventions in primary care settings. We found no data from any country in the Western Pacific Region, including Lao PDR, on gaps in midwifery regulatory policies. We searched Medline with the keywords, midwife and regulation; and sources in Lao PDR including published government documents.

**Added value of this study**

This study developed a systematic method for quantifying key RMNCAH interventions in the essential health service package (EHSP) that must be delivered by midwives in primary healthcare settings and determining whether regulatory policies support the delivery of these interventions. The analysis found that regulations in Lao PDR legally supported midwives to deliver only 8 out of 39 (20.5%) RMNCAH interventions in primary care settings without a physician. This analysis allowed the Ministry of Health and Lao Midwife Association to revise the regulatory scope of practice to align with the EHSP. After the revision, an additional 29 required midwifery interventions were legally approved, resulting in regulatory support for 37 of 39 essential interventions (94.9%). This study has contributed to advancement of developing a systematic regulatory gap analysis, which enables practical and targeted regulatory revisions for better policy alignment.

**Implications of all the available evidence**

This quantitative analysis found significant regulatory gaps for services midwives are expected to provide at the primary care level. Similar gaps are likely to exist for other service areas, health worker cadres and in other countries. The method should be considered as an approach to strengthening regulatory support for primary care workers and reaching vulnerable populations in other countries. Regulatory support is only one factor required to enable practice. Other factors limiting midwifery practice in health facilities must be better understood and addressed, including the content and quality of education, health system gaps, and socio-cultural, economic and professional barriers.

## Introduction

Achieving universal health coverage (UHC) with quality health services (Sustainable Development Goal Target 3.8) is central to reducing health inequities and improving health outcomes.^1^^,^^2^ Defining essential health services supports countries to achieve UHC by providing a framework to prioritize source allocation and equip health workforce to deliver those services.^3^ As the world continues to suffer from shortages of human resources, ensuring efficient use of existing human resources for service delivery is crucial for achieving UHC.^4^, ^5^, ^6^ However, shortfalls in access, quality, efficiency, and equity of essential services have been documented extensively in countries across income levels.^7^^,^^8^

Lao PDR, a country of 7.5 million people in Southeast Asia, has experienced significant improvements in maternal and child health outcomes. Between 2000 and 2020, maternal mortality fell from 579 to 126 per 100,000 live births and under five mortality from 107 to 44 per 1000 live births.^9^^,^^10^ It is among the few countries to achieve both Millennium Development Goals 4 and 5. Although population coverage of key interventions along the continuum of care for women and children has steadily improved, quality of primary care services remains a challenge. For example, 78.4% of women attended at least 1 antenatal care in 2017 but only 40.2% had a blood sample taken at least once during the pregnancy. Similarly, 64.4% of livebirths were assisted by a skilled birth attendant in 2017, but only 45.9% of the newborns received a health check following the birth.^11^ Improving healthcare quality at primary care level is therefore a priority and an essential component of the national strategy and action plan on reproductive, maternal, newborn, child, and adolescent health (RMNCAH) 2016–2025.^12^

An effective health care workforce is essential for reaching remote populations and improving care quality. Midwives and nurses often play significant role in providing primary RMNCAH services.^13^ However, gaps are widespread in their availability, distribution, skills mix, productivity and performance, resulting in uneven care quality and coverage.^6^^,^^14^ To narrow this gap for maternal and child health, it is crucial to empower midwives and nurses through comprehensive investment in regulation, education and systems investments to improve service delivery.^15^ However, midwifery regulatory system does not often align with international standards.^16^, ^17^, ^18^, ^19^ Further, due to the lack of investment in certified registration, effective regulatory bodies, or professional associations, licensing to ensure practice quality is often not adopted or enforced.^20^^,^^21^ Insufficient regulatory support creates particular challenges at primary care level where midwives and nurses are the main service providers. When no physician is present, midwives and nurses become the only maternal and child health providers available and may be called upon to deliver care interventions without legal protection. Even at health facilities with physicians, competing priorities limit the ability of physicians to provide oversight including working in private practice, performing government functions or high patient loads.^22^^,^^23^ Midwives and nurses are therefore often required to deliver care alone. There are anecdotal reports in Lao PDR, that medical staff increasingly have private sector positions that make them less available to work at government facilities. Addressing these challenges requires coordinated policy responses to ensure that midwives and nurses operate within an enabling environment and receive the required system supports.^24^^,^^25^

In Lao PDR, the Essential Health Service Package (EHSP) was first endorsed in 2018 to allow financial and human resources to be allocated to service priorities under the Health Sector Reform Strategy and Framework (2016), which calls for medical staff shortages to be filled by midwives and nurses.^26^^,^^27^ Priority was given to midwives because of the national focus on improving the availability of skilled birth care to reduce the high maternal and newborn mortality, especially in remote areas. Thus, Lao PDR invested in midwifery education, through developing and strengthening midwifery diploma education and establishing a midwife association, with the goal of increasing the proportion of health centers with at least one midwife.^27^, ^28^, ^29^ However, no data were available to assess whether the current regulatory framework on midwifery practice supported midwives to deliver essential services without a physician, defined in Lao PDR as a medical doctor or a medical assistant (a cadre with 3 years of education who can provide all basic medical services including diagnosis, prescription of medicines, treatment, counselling and issuing medical certificate, at all facility levels). This information was especially important in health centers, only 75.4% of which have a physician, with midwives or nurses the only available provider of RMNCAH services.^30^

To address this gap, a policy analysis to identify regulatory gaps was undertaken by the Ministry of Health (MoH) and WHO in Lao PDR. This study aimed to identify essential RMNCAH interventions that were expected to be provided by midwives without the presence of a physician, map whether current regulatory standards supported delivery of these interventions and use data to update these regulatory standards.

## Methods

Document search and review were conducted from September to December 2021 as a part of policy reviews to support the national strategy and action plan on RMNCAH 2016–2025.

### Document search

Consultations were conducted with the national RMNCAH committee to identify existing national standards, guidelines and in-service training materials in July 2021. The committee consists of 5 sub-committees on Reproductive, Maternal, Newborn, Well Child, and Sick Child. Membership includes the MoH and professional associations representing midwifery, obstetrics, and pediatrics. One consultation took place with each sub-committee. Then twenty-three development partners supporting the committee were consulted through email to add potentially missing standards or guidelines that had been adopted widely (Supplement). Documents were included if they described clinical care or service delivery protocols or practices required to deliver essential RMNCAH interventions included in the national Essential Health Service Package (EHSP) and were endorsed by MoH or approved by recognized professional associations after 2007 (the year the Ministerial Decision on Nursing and Midwifery Regulations was established in Lao PDR).^31^

### Document review for regulatory gap analysis

Identified national standards and guidelines were reviewed using steps 1–3 below to identify interventions and care tasks that were expected to be provided by midwives without a physician. The Lao PDR Ministerial Decision on Guidelines for the Scope of Midwifery Practice (Lao Scope of Practice) was mapped against the list of interventions and care tasks to identify gaps in the final step (step 4) (Table 1). The Lao Scope of Practice is the national standard used in The Ministerial Decision on Nursing and Midwifery Regulations as the basis for detailed descriptions of services that midwives are legally allowed to provide.^31^^,^^32^

**Table 1:** RMNCAH interventions and care tasks in the national EHSP fully supported by the Lao PDR Scope of Midwifery Practice, before and after revision, Lao PDR, 2021.

Service area	Number of interventions and care tasks in the EHSP that should be provided by midwives alone	Interventions and care tasks fully supported by the Lao Scope of Midwifery Practice, n (%)	
		Pre revision	Post revision
Adolescent/pre-pregnancy/sexual health	Interventions, 9 Care tasks, 143	1 (11%) 101 (70.6%)	9 (100%) 143 (100%)
Safe Abortion	Interventions, 4 Care tasks, 120	0 (0%) 59 (49.2%)	2 (50%) 101 (86.7%)
Antenatal care	Interventions, 10 Care tasks, 177	1 (10%) 111 (62.7%)	10 (100%) 177 (100%)
Intrapartum care	Interventions, 7 Care tasks, 214	3 (42.9%) 160 (74.8%)	7 (100%) 214 (100%)
Postpartum/postnatal care	Interventions, 2 Care tasks, 19	1 (50%) 18 (94.7%)	2 (100%) 19 (100%)
Newborn care	Interventions, 3 Care tasks, 54	2 (66.7%) 47 (87.0%)	3 (100%) 54 (100%)
Child care (for well and sick children)	Interventions, 4 Care tasks, 373	0 (0%) 209 (56%)	4 (100%) 373 (100%)
Total	Interventions, 39 Care tasks, 1100	8 (20.5%) 705 (64.1%)	37 (94.9%) 1081 (98.3%)

### Step 1: define essential RMNCAH interventions and care tasks in Lao PDR

The national EHSP was used to identify essential RMNCAH interventions in Lao PDR.^26^ RMNCAH interventions were defined as evidence-based practices or treatments demonstrated to prevent illness and death and improve outcomes across the lifecycle including pre-pregnancy, pregnancy, birth and postpartum, newborn, child and adolescence). National clinical standards, guidelines and training materials identified in the document search were used to define detailed care tasks for interventions in the EHSP. Care tasks were defined as individual clinical actions required to deliver an intervention. Care tasks were organized into previously defined practice categories adapted from globally accepted international norms^24^^,^^33^: (1) history taking, (2) physical examination, (3) ordering tests, (4) conducting tests, (5) interpreting tests, (6) diagnosis, (7) education, counselling, and advice for uncomplicated cases, (8) preventative prescription and administration of medicines/other agents, (9) non-pharmacological interventions for uncomplicated cases, (10) education, counselling, and advice for cases with complications, (11) curative prescription, (12) administration of medicines, and (13) non-pharmacological intervention for cases with complications. All interventions and care tasks were organized along the lifecycle including pre-pregnancy, pregnancy, birth and postpartum, newborn, child and adolescence.

### Step 2: determine whether EHSP interventions were consistent with the internationally recommended standards

The RMNCAH interventions currently recommended in Lao PDR identified in Step 1 were mapped against evidence-based internationally recommended intervention packages and standards of care, using the most up-to-date evidence synthesis at the time of analysis.^3^

### Step 3: identify interventions and care tasks that were expected to be provided by midwives without a physician

The standard list of interventions identified in Step 1 was shortened to include only those interventions and care tasks that midwives are expected to provide without a physician based on internationally used competencies and scope of practice of midwives.^24^^,^^33^ Interventions required in the EHSP at health centers, the first-level facility in Lao PDR, were also included even if not recommended in the international practice standards to ensure that the final list of interventions was consistent with what the EHSP requires midwives to provide without physician’s presence.^26^

### Step 4: identify midwifery regulatory policy gaps (before and after revision of the regulation)

Interventions and care tasks included in the Lao Scope of Practice were compared with those that midwives should provide without a physician identified in Step 3. If the Lao Scope of Practice included both an intervention and all supporting care tasks required to deliver the intervention, then this intervention was classified as legally allowed by regulations. The Lao Scope of Practice categorizes service delivery into four areas:

(1) Care tasks that midwives can practice on their own in all settings or in remote settings;
(2) Care tasks that midwives can practice only with a physician’s prescription;
(3) Care tasks that midwives can practice only in the presence of a physician; and
(4) Care tasks that midwives can practice in case of emergency.

As this analysis focused on interventions that can be delivered by midwives without a physician, interventions and care tasks in categories 1 and 4 were used for analysis. Step 4 was done twice: before and after the MoH revised the regulatory policy.^34^ The Lao Scope of Practice was updated in November 2021 using findings from the gap analysis completed in October 2021. The MoH led a two-week multi-stakeholder workshop to identify gaps, review evidence and revise policies with the Nursing and Midwifery Board of the Healthcare Professional Council, the Lao Midwife Association, UNFPA and WHO.

### Ethics approval

This study obtained an ethical approval from the National Ethics Committee for Health Research in Lao PDR (Submission ID: 2021.52) and the University of Tokyo (Registration number: 2021213NI). In Lao PDR, MoH classified the consultations and document review as a part of the implementation of the national strategy and action plan on RMNCAH 2016–2025. No informed consent was necessary from individuals to identify existing national standards and guidelines.

### Role of the funding source

The funding was used for operational cost of the analysis. The funder had no role in the study design, data collection, data analysis, data interpretation, or writing of the report.

## Results

### Document search and step 1: define essential RMNCAH interventions and care tasks in Lao PDR

The national EHSP contained 100 health interventions, of which 47 were related to RMNCAH. The document review yielded 18 national standards, guidelines, and national standard training materials (Box 1), from which 1227 care tasks were identified as necessary to deliver the 47 RMNCAH interventions in the EHSP.

Box 1 **National standards and guidelines identified for defining essential RMNCAH interventions, Lao PDR, 2021**

• National Adolescent and Youth Friendly Service Guideline (2018). Available at: https://drive.google.com/file/d/1uDY_rGJ3MiyNolTIFrP9dde2lAmQ1Ibd/view
• National Adolescent and Youth Friendly Service training material (2017)
• Job aid for Adolescent Youth Friendly Service providers (2019). Available at: https://drive.google.com/file/d/1H2Mw442LLzDbPeXNpidRMSQqDQyj4kYu/view
• Family Planning A Global Handbook for Providers Third Edition (2018). Available at: https://drive.google.com/file/d/1JX4S3IJNKO2ecUUT-7sVxQT4Pav3FIx8/view
• Family Planning Training Package (2016).
• Lao national STI guideline: manual of treatment for sexual transmitted infection diseases (2018). Available at: https://drive.google.com/file/d/1wNmTWRITjxelonx8Wp0OUpJxH6VWdy2n/view
• Unsafe Abortion Prevention and Care Practical Guideline for Health Workers (2016). Available at: https://drive.google.com/file/d/10jL_gXHUHzwmYMr-sndGCCvDtF4Jn0oK/view
• The training on prevention of unsafe abortion (2019)
• Guidelines and Reference book on Antenatal Care and Postnatal Care (2018). Available at: https://drive.google.com/file/d/1zzJzSA7xntRHinXW4MrNizPli08Hz3MQ/view
• Antenatal Care coaching (training material) (2020)
• Participant manual on health center training on integrated essential MNCH services (2021)
• Pocketbook on Essential Care for Childbirth and Maternal Complications (2020). Available at: https://drive.google.com/file/d/1AGgLXsdpoYkF7VIWj06PjtVa1jWGQDGq/view
• Intrapartum Care/Emergency Obstetric Care training module material (2020)
• Early essential newborn care (EENC): clinical practice pocket guide (2014). Available at: https://drive.google.com/file/d/1imH75oVuoDUgknSZrIkwYkfBtHMjvvne/view
• Introducing and sustaining EENC in hospitals: Kangaroo Mother Care for preterm and low-birthweight infants (2018). Available at: https://drive.google.com/file/d/1E4Gym8RfsgT4UCLAF-sMZ_x3gapuBe5b/view
• Integrated Management of Childhood Illness Chartbook (2018). Available at: https://drive.google.com/file/d/1DnVAzpSz-MKM2FBRHeR22BZeDAfzsOWm/view
• Community Integrated Management of Childhood Illness Job aid (2019)
• Pocketbook of Hospital Care for Children 2nd edition (2013). Available at: https://drive.google.com/file/d/158e8yWbuF1JtikTihIXbpbATygkFemR3/view

### Step 2: determine whether EHSP interventions were consistent with internationally recommended standards

The national EHSP does not include the management of complications of female genital mutilation (FGM), currently a recommended RMNCAH intervention for areas where this is a public health problem. To our knowledge, FGM has not been reported in Lao PDR. Otherwise, all currently recommended international RMNCAH interventions were included in the EHSP.

### Step 3: identify interventions that were expected to be provided by midwives without a physician

Based on internationally used competencies and scope of practice,^24^^,^^33^ midwives should be able to provide 36 of the 47 (76.6%) RMNCAH interventions in EHSP without a physician. An additional three were expected to be provided by midwives at health centers without a physician in the context of Lao PDR. The three added interventions were: management of complications following abortion, treatment of syphilis for mothers and newborns, and integrated management of newborn and childhood illnesses. In total, 39 interventions and 1100 care tasks were identified for provision by midwives without a physician. The excluded interventions were treatment of pre-cervical cancer by cryotherapy and by Loop electrosurgical excision procedure, advanced complication management following abortion, induction of labour using oxytocin, management of maternal complications including comprehensive emergency obstetric care such as caesarean section, neonatal respiratory distress syndrome management using CPAP, management of newborn with other complications, and advanced sick childcare. These interventions were not expected to be provided by midwives without a physician’s presence either by the international scope of midwifery nor by the national EHSP.

### Step 4: identify midwifery regulatory policy gaps (before and after revision of the regulation)

Of the 1100 care tasks and 39 RMNCAH interventions that should be provided by midwives, 64.1% (705/1100) and 20.5% (8/39) respectively were included in the Lao Scope of Practice (Table 1). Thirty-one of 39 (79.5%) interventions were not allowed for provision by midwives alone because the interventions required one or more care tasks to be performed by a physician or in the presence of a physician. Of the 31 interventions, 16 required ordering and conducting diagnostic tests (e.g. blood testing for anaemia during antenatal care), 12 required making a clinical diagnosis, 26 required prescribing and administering medicines (e.g. administering contraceptives or prescribing medicines for integrated management of childhood illness), and 7 required the use of non-pharmacological interventions.

### Revision of regulatory standards

After the revision, 1081 of 1100 (98.3%) care tasks and 37 of 39 (94.9%) RMNCAH interventions were approved for provision by midwives alone (Table 1).

General practice categories were added into the revised Lao Scope of Practice. These included ordering and conducting tests, initial diagnosis (identification of clinical conditions requiring treatment or referral), preventive prescription, and administration of medicines/other agents. Furthermore, curative prescription, administration of medicines, and non-pharmacological interventions also became legally permitted for midwives without a physician in case of emergency, first-line management, and pre-referral care.

As a result, several interventions could be added including: use of long-acting reversible contraceptives; pregnancy care including prevention of mother to child transmission of syphilis and HIV, anaemia management, management of hypertension and gestational diabetes mellitus; basic emergency obstetric care including management of pre-eclampsia, eclampsia, postpartum hemorrhage and sepsis; well child services; and primary care for the sick child.

Surgical abortion management and medical abortion after 12 weeks remained unallowed. While Manual Vacuum Aspiration (MVA) for management of incomplete abortion was added, MVA for complete surgical abortion management was not. These were not added into the revised Lao Scope of Practice because the Lao Midwife Association deemed the current technical capacity of midwives and teaching staff to be insufficient to provide these interventions safely (Table 2, Table 3).

**Table 2:** RMNCAH interventions in the national EHSP supported by the Scope of Midwifery Practice before revision, Lao PDR, 2021.

**Table 3:** RMNCAH interventions in the national EHSP supported by the Scope of Midwifery Practice after revision, Lao PDR 2021.

## Discussion

This study found that at baseline, only 8 of 39 (20.5%) essential RMNCAH interventions could legally be provided by midwives without a physician in Lao PDR. Use of a systematic process to review RMNCAH regulations against national essential services and standards allowed the MOH to quantify these regulatory gaps for the first time. Revisions to the Lao Scope of Practice resulted in 37 of 39 (94.9%) essential RMNCAH interventions being legally approved for delivery by midwives working on their own. This resulted in a significant policy alignment and provided a foundation on which subsequent efforts to improve the quality of care provided by midwives can be built.

At baseline, only 20.5% of RMNCAH interventions were allowed by the Lao Scope of Practice compared to 64.1% of care tasks, as shown in Step 4. This was because the delivery of interventions often involves multiple care tasks. Only when all care tasks required to deliver each intervention were included in the regulation, was the intervention legally allowed. For example, provision of long-acting reversible contraceptives requires history taking, physical exam, education, counselling and advice (uncomplicated cases), testing (order, conduct, and interpret), and preventive prescription. As the Lao Scope of Practice did not include testing (order and conduct) or preventative prescription at baseline, this intervention was unsupported by the regulations. This example highlights the benefits of this analysis. The specificity created by breaking interventions into required care tasks helped systematic identification of legally unsupported interventions due to practice categories that were not covered in the Lao Scope of Practice. By simply adding these practice categories into the Lao Scope of Practice, many interventions became legally allowed to be provided by midwives alone.

Global evidence suggests that midwives working in health centers require higher autonomy compared to those in hospitals.^35^ Various factors may contribute to their ability to practice autonomously, including their technical proficiency and their work environment. However, without regulatory support for autonomous provision of essential primary care services, midwives have no legal protection, are potentially liable for negative health outcomes, and are less likely to be supported by managers and other staff to perform these roles, which lead to suboptimal quality of care. The revised Lao Scope of Practice effectively provides a legal framework for clinical care provided by midwives, thereby establishing the basis for standards and regulations to support this framework. This analysis contributed to better understanding the extent to which regulations supported autonomy of midwifery practices, on which research has been scarce. However more research is needed on midwifery practice barriers in primary care settings in Lao PDR, how improved regulation can be used to address these barriers, and how the role of midwives can be changed and integrated into routine practice.

The gap analysis was possible because the Lao government had defined essential interventions in the EHSP and detailed care tasks for each intervention in the existing national guidelines. Steps 1 to 3 were crucial for the main gap analysis in step 4. Identifying essential interventions and breaking them into care tasks in Step 1 enabled detailed mapping of the content of the Lao PDR Scope of Practice, which was required to identify which interventions were legally allowed by midwives to provide without a physician (Supplement). Step 2 showed that the national EHSP was aligned with international standards with context consideration. Only one of 43 recommended RMNCAH interventions—management of complications following FGM—was not in the national EHSP.^3^ Currently, there are no known reports of FGM from Lao PDR and removal of this intervention from the EHSP was a reasonable decision based on available data.^36^^,^^37^ However, in some settings there may be significant differences between recommended interventions and country standards; therefore Step 2 can be used to update nationally recommended interventions which can then serve as the reference for the following steps. Step 3 further cuts down the list to interventions that are expected to be provided by midwife without a physician, as those are the interventions that need to be supported in the Lao Scope of Practice.

Based on the gap analysis the MoH, the Nursing and Midwifery Board of the Healthcare Professional Council, and other stakeholders updated the national midwifery regulations, which MoH immediately endorsed. Gaps between evidence and policies are often reported, however such rapid revision and adoption does not always occur.^38^^,^^39^ Three potential reasons for this successful evidence-based policy decision were identified. First, the issue of misalignment between regulations and practice requirements was already recognized widely before quantification. National facility-based quality of care assessments on RMNCAH had identified barriers to effective practice at the primary care level including discrepancies between what midwives were expected versus allowed to provide. Better alignment of workforce regulation and education standards to the EHSP was subsequently articulated as an action point in the national strategy.^12^ Second, effective coordination among national stakeholders allowed joint engagement at all stages of the analysis and revision. Involvement of the MoH, the Nursing and Midwifery Board of the Healthcare Professional Council, the Lao Midwife Association and the National RMNCAH Committee ensured a common understanding of the purpose and technical basis of the analysis, and therefore rapid consensus on revisions. Third, the analysis enabled quantification, visualization and clarification of practical implications of gaps in a way that had not been available previously, which helped decision-makers to better understand the needs.

This analysis and the subsequent revision of regulations are expected to facilitate the provision of essential RMNCAH services by empowering and protecting midwives through a regulatory framework. Deploying midwives without adequate regulation is neither effective nor safe.^17^^,^^40^ While the analysis contributed to better policy alignment, improving access to and quality of RMNCAH services will require wider investments in the overall service delivery network and systems required to support care delivery such as the availability of essential equipment and medicines.^40^ Further, other factors may limit the ability of midwives to practice effectively, including social inequality, inadequate pay to meet the basic cost of living, unsafe or poor working, living and social conditions, and physical and sexual abuse.^41^^,^^42^ More data are needed in Lao PDR on factors which contribute to and limit effective midwifery practice. While disseminating, monitoring, and supporting compliance with the regulatory framework is critical, it is likely that sustained improvements in midwifery practices will require the development of enabling environments to overcome other socio-economic and practice barriers.

The MoH in Lao PDR will coordinate follow-up actions with key midwifery stakeholders involved in the regulatory gap analysis, and provincial and district authorities. Four areas are proposed for on-going system strengthening. First, to establish a mechanism for making regulatory review routine. This will allow the list of essential RMNCAH interventions to be rapidly updated based on emerging global evidence and national priorities, and reflected in regulations. Second, to develop midwifery policies to support sustained practice norms and standards. These include updating the midwifery curriculum to align with RMNCAH interventions in the EHSP, regularly updating knowledge and skills of teachers, establishing a post-licensure education program, board certification and license renewal criteria, and developing legislation and regulation on continuous professional development. Third, to better understand reasons for midwifery primary care practice gaps. Systems interventions to improve practices are part of an ongoing primary health care quality improvement initiative. Finally, to conduct a regulatory gap analysis and update of regulations for nurses and other cadres that also provide RMNCAH primary care services to allow the further development of a comprehensive and coordinated human resource development plan.

### Limitations

This analysis maps the midwifery practice regulations against RMNCAH interventions and care tasks included in the EHSP and 18 national standards and guidelines identified through document search. However, complete mapping of care tasks was not possible when identified national standards or guidelines did not provide detailed descriptions of clinical content. For example, care tasks required for delivery of postnatal care were not described in detail in existing documents. It was therefore impossible to assess whether regulations supported all care tasks required to provide postnatal care.

This analysis maps the regulatory framework against RMNCAH interventions in the EHSP, whose scope is limited to individual clinical care. Thus, it has not captured standards of midwifery practice beyond clinical settings.^43^ For example, according to International Confederation of Midwives, midwives are expected to facilitate teamwork with other care providers and community groups; and to play a community outreach role to improve access to essential services.

In addition, the analysis focused on identifying gaps in the Lao Scope of Practice, but not how midwifery roles and responsibilities overlap with those of other healthcare professionals such as doctors and nurses. This is important to ensure efficient use of human resources, appropriate staff allocation and task division.^44^ Therefore an ongoing process is required to ensure that the skill mix of different cadres are complementary and contribute most efficiently to improving access to care. A cost-effectiveness analysis is needed to determine the relative cost and outcome benefits of primary health care provided by midwives and other mid-level care providers, relative to doctors or other care models.

Lastly, it should be noted that the service delivery model in Lao PDR is heavily dependent on public healthcare facilities, which provide primary care services at low cost for most of the population through the National Health Insurance scheme. This makes it more straightforward to standardize essential services by facility type. The model used in this analysis may require modification when applied to countries with a more complex mix of services and facility types.

### Conclusion

This study demonstrated a systematic approach to identifying regulatory gaps for the delivery of essential RMNCAH interventions by midwives in Lao PDR and taking action to address them. It resulted in a 74.4% point improvement in the proportion of interventions that midwives can provide on their own supported by the Lao Scope of Practice. This improvement was achieved by adding practice categories that were previously not included in regulations, such as ordering and conducting diagnostic tests and preventive prescription and administration into the Lao Scope of Midwifery Practice. Since midwives are key providers of primary care services in Lao PDR, this regulatory change was fundamental for improving pre-service training curriculums and standards, regulations and licensing, and ultimately the quality of RMNCAH practice at primary care facilities. This method is applicable to other service areas and cadres and should be considered as an approach for strengthening human resources availability and capacity; and expanding access to primary health care for all populations.

## Contributors

Kubota Shogo: conceptualization, methodology, data verification, writing (original draft).

Moe Ando: methodology, investigation, data curation, data verification, writing (review and editing).

John Murray: methodology, writing (review and editing).

Sengmany Khambounheuang: writing (review and editing).

Khampasong Theppanya: supervision, writing (review and editing).

Phouvanh Nanthavong: project administration, writing (review and editing).

Chankham Tengbriacheu: writing (review and editing).

Malouny Sisavanh: writing (review and editing).

Thongchan Khattiyod: investigation.

Daisuke Asai: writing (review and editing).

Howard Sobel: methodology, writing (review and editing).

Masamine Jimba: writing (review and editing).

## Data sharing statement

A list of RMNCAH Interventions required to be delivered by midwives without physicians and status of regulatory support by the midwifery scope of practice before revision in Lao PDR is available in the Web Annex.

## Declaration of interests

There is no conflict of interest to disclose.

## Appendix A Supplementary data

Web Annex 1 and 2

Translated abstract
