# Supplementary material for: A regulatory gap analysis of midwifery to deliver essential reproductive, maternal, newborn, child and adolescent health services in Lao People’s Democratic Republic
Source: Lancet Reg Health West Pac. 2023 Dec 5;43:100960. doi: 10.1016/j.lanwpc.2023.100960 (PMC10749282; doi:10.1016/j.lanwpc.2023.100960)
Supplement: Web Annex 1 and 2 [file mmc1.docx]

**Supplementary materials**

# **Web Annex 1: The list of consulted development partners to identify existing RMNCAH national standards, guidelines, and in-service training materials in July 2021**

| 1. The World Health Organization (WHO) 2. The United Nations Joint Programme 3. The United Nations Population Fund (UNFPA) 4. The United Nations Children's Fund (UNICEF) 5. The Swiss Red Cross 6. The Luxembourg Development Cooperation Agency (LuxDev) 7. The Safe the Children International (SCI) 8. Population Services International (PSI) 9. Korea Foundation for International Healthcare (KOFIH) 10. CARE International 11. The World Bank 12. The Japan International Cooperation Agency (JICA) 13. World Vision International (WVI) 14. Plan International 15. The European Union (EU) 16. The United States Agency for International Development (USAID) 17. Humanity and Inclusion (HI) 18. Gavi, the Vaccine Alliance (GAVI) 19. Clinton Health Access Initiative (CHAI) 20. The Service Fraternel d’Entraide (SFE) 21. The Adventist Development and Relief Agency (ADRA) 22. Lao Friends Hospital for Children 23. Medecins du Monde |
| --- |

**Web Annex 2: RMNCAH Interventions in the national Essential Health Service Package (EHSP) required to be provided by midwives alone and the status of regulatory support by the Lao PDR Scope of Midwifery Practice (2009) before revision, Lao PDR, 2021**

| **Service area** | | **Regulatory support status Fully supported (Yes / No)** | **Practice categories not fully supported by regulation*1** | | | | | |
| --- | --- | --- | --- | --- | --- | --- | --- | --- |
| **#** | **Essential** **RMNCAH Interventions in the national EHSP** |  | **Test (Order/Conduct)** | **Diagnosis** | **Preventative prescription and administration** | **Curative prescription / Administration of medicine** | **Non-pharmacological intervention (uncomplicated)** | **Non-pharmacological intervention (cases with complications)** |
| **Adolescent /Pre-pregnancy/Sexual health** | |  | | | | | | |
| 1 | Health education, promotion and counseling for improved health behavior and necessary care seeking behavior in the RMNCAH areas; including reproductive and sexuality; continuum of care during pregnancy, childbirth, postpartum, and childcare, prevention and management of gender-based violence and child abuse. | Yes |  |  |  |  |  |  |
| 2 | Tetanus immunization to women of reproductive age and adolescents to protect neonatal tetanus | No |  |  |  |  |  |  |
| 3 | Weekly iron & folic acid supplementation for women of reproductive age | No |  |  |  |  |  |  |
| 4 | Short acting contraceptive 1) Condom 2) Oral contraceptives 3) Injectable | No |  |  |  |  |  |  |
| 5 | Long-acting reversible contraceptive 1) Implant 2) IUD (intrauterine device) | No |  |  |  |  |  |  |
| 6 | Permanent contraceptive  1) Vasectomy 2) Tubal ligation | No |  |  |  |  |  | *2 |
| 7 | STI management: Syphilis, Gonorrhea, Chlamydia (including pelvic infection disease) *3 1) Detect suspected case 2) Diagnosis 3) Treatment | No |  |  |  |  |  |  |
| 8 | HIV Counselling and Testing (HCT) *4: Client initiative counseling and testing (CICT) and Provider initiative counselling and testing (PICT) | No |  |  |  |  |  |  |
| 9 | Cervical cancer screening for women (e.g. Visual inspection of cervix with acetic acid) | No |  |  |  |  |  |  |
| **Safe Abortion** | |  | | | | | | |
| 10 | Counselling for women with unplanned, mistimed or unwanted pregnancies, including abortion services | No |  |  |  |  |  |  |
| 11 | Medical abortion management 1) Medical abortion 2) Routine post-abortion follow-up, including post-abortion contraceptives | No |  |  |  |  |  |  |
| 12 | Surgical abortion management *5 1) Surgical abortion 2) Routine post-abortion follow-up, including post-abortion contraceptives | No |  |  |  |  |  |  |
| 13 | Complication management followed by abortion 1) Ongoing pregnancy 2) retained products/incomplete abortion 3) Infection 4) Haemorrhage | No |  |  |  |  |  |  |
| **Antenatal Care** | |  | | | | | | |
| 14 | Basic routine antenatal care (ANC)  1) Body weight check  2) Blood pressure check and screening of sign of severe preeclampsia  3) History taking  4) Screening of anemia by checking physical anemia signs  5) Screening of risk of preterm birth  6) Fundal height check  7) Abdominal palpation (Leopord maneuver)  8) Fetal heart check  9) Check oedema and breasts  10) Health education and counselling (e.g. Birth preparedness)  11) Calcium supplement provision  12) Gestational diabetes mellitus risk factor screening with past and family history, BMI (>30kg/m2). When positive in screening, glycosuria on dipstick testing  13) Provide counselling on STI including syphilis and HIV/ AIDS  14) Testing of HIV/ AIDS  15) Testing of STI (syphilis)  16) Daily Iron and folate acid to prevent maternal anaemia, puerperal sepsis, low birth weight, and preterm birth  17) Td immunization to pregnant women at least two doses if no previous Td immunization and one dose for subsequent pregnancies  18) Distribute Long-lasting insecticidal nets (LLIN) to pregnant women at ANC in strata 2b and 3 districts defined by the Ministry of Health | No |  |  |  |  |  |  |
| 15 | Gestational age specific ANC 1) Screening of anaemia (Hb/Hct check) 2) Provide deworming for pregnant women after the first trimester 3) Ultrasound scan at ANC before 24 weeks of gestation to estimate gestational age | No |  |  |  |  |  |  |
| 16 | ANC package during outreach service 1) Body weight check  2) History taking  3) Sign of pre-eclampsia  4) Blood pressure check  5) Screening of anemia (check anemia signs)  6) Fetal heart check  7) Health education and counselling  8) Calcium supplement provision  9) Daily Iron and folate acid to prevent maternal anaemia, puerperal sepsis, low birth weight, and preterm birth  10) Td immunization to pregnant women at least two doses if no previous Td immunization and one dose for subsequent pregnancies | No |  |  |  |  |  |  |
| 17 | ANC in community Assist pregnant women at community level: Individual health education, detecting antenatal danger signs and refer to health facility, birth and emergency preparedness (e.g. plan for emergency transportation, prepare medical documents, or payment) | Yes |  |  |  |  |  |  |
| 18 | Anemia management at ANC 1) Treatment (double dose of iron & folic acid) | No |  |  |  |  |  |  |
| 19 | Hypertension management at ANC: Antihypertensive drugs to treat high blood pressure | No |  |  |  |  |  |  |
| 20 | Low dose aspirin prophylaxis for women at moderate and high-risk of pre-eclampsia and eclampsia | No |  |  |  |  |  |  |
| 21 | PMTCT-Syphilis management 1) Treatment of Syphilis for mother 2) Treatment of Syphilis for newborn | No |  |  |  |  |  |  |
| 22 | PMTCT-HIV management *6 | No |  |  |  |  |  |  |
| 23 | Gestational Diabetes mellitus management at ANC *7 | No |  |  |  |  |  |  |
| **Intrapartum Care** | |  | | | | | | |
| 24 | Routine intrapartum care 1) Monitoring vaginal delivery 2) Provide a positive childbirth experience 3) Active management of the third stage of labour: Routine administration of a uterotonic after childbirth to prevent postpartum haemorrhage, controlled cord traction (optional), uterine massage (if the uterus is not well contracted) | No |  |  |  |  |  |  |
| 25 | Assisted vaginal delivery | Yes |  |  |  |  |  |  |
| 26 | Preterm labour management  1) Corticosteroids to prevent respiratory distress syndrome 2) Magnesium sulfate for fetal neuroprotection in preterm babies | No |  |  |  |  |  |  |
| 27 | Augmentation for prolonged labour | No |  |  |  |  |  |  |
| 28 | Manual removal of placenta, remove retained products (MVA) | Yes |  |  |  |  |  |  |
| 29 | Basic Emergency obstetric care (BEmOC): Management of maternal complications *8 1) sepsis management (Administer parenteral antibiotics for cases with indication) 2) Postpartum haemorrhage management (uterotonic drugs for management of PPH) 3) pre-eclampsia/eclampsia management (parenteral anticonvulsants for pre-eclampsia and eclampsia (i.e. magnesium sulphate)) | No |  |  |  |  |  |  |
| 30 | Care for unplanned home delivery 1) Minimum maternal and newborn care for unplanned home delivery (e.g. cord care, immediate and thorough drying, immediate skin-to-skin contact, initiation of exclusive breastfeeding) 2) Detect danger signs for mother and baby during unplanned home deliveries, and refer to health facility | Yes |  |  |  |  |  |  |
| **Postpartum/ Postnatal Care** | | |  | | | | | |
| 31 | Routine PNC for mothers and babies  1) Regular assessment for mothers: general well-being, vital signs (blood pressure, temperature, heart rate), fundal height, uterine contraction, vaginal bleeding, check perineal wound/c-section wound, lochia, uterine involution, urine void, bowel function, breast condition, assessment and counselling on breastfeeding progress, review of emotional well-being, observation for domestic abuse 2) Regular assessment for newborn (weight, feeding, history of convulsions, fast breathing, chest in-drawing, spontaneous movement, fever, low body temperature, jaundice <24hrs, yellow palms and soles, Dry cord care and assessment of signs of cord infection, urination, passing stool, pallor, skin/eye infection) 3) counselling on physiological recover, newborn care, danger signs for mothers and newborns, nutrition, hygiene, malaria protection, mobilisation, family planning, breastfeeding, immunization for newborns 4) Provide Iron and folic acid supplementation for postpartum/ lactating women | No |  |  |  |  |  |  |
| 32 | Community PNC: Detecting postpartum danger signs and refer to health facility | Yes |  |  |  |  |  |  |
| **Newborn Care** | |  | | | | | | |
| 33 | Routine newborn care: 1) Early essential newborn care (immediate and thorough drying, immediate skin-to-skin contact, delayed cord clamping, dry cord care, initiation of exclusive breastfeeding, eye prophylaxis) 2) Routine immunization for newborns according to the national guidelines (BCG vaccine and Hepatitis B vaccine 1st dose) | Yes |  |  |  |  |  |  |
| 34 | Neonatal resuscitation | Yes |  |  |  |  |  |  |
| 35 | Kangaroo Mother Care for preterm and low-birth weight infants | No |  |  |  |  |  |  |
| **Child Care (for well and sick children)** | |  | | | | | | |
| 36 | Well child services at health facility 1) Routine immunisation of children (BCG, DPT-HepB-Hib, Polio (IPV/OPV), Measles-Rubella, PCV, JE) 2) Vitamin A and deworming for under 5 children 3) Screening and counselling on breastfeeding/complementary feeding 4) Screening and counselling on early childhood development 5) Growth monitoring and counselling | No |  |  |  |  |  |  |
| 37 | Well child services at outreach 1) Routine immunization of children (BCG, DPT-HepB-Hib, Polio (IPV/OPV), Measles-Rubella, PCV, JE) 2) Vitamin A and deworming for under 5 children 3) Screening of nutrition status with MUAC for children under 5 years of age | No |  |  |  |  |  |  |
| 38 | Primary care for sick child  1) Growth assessment/nutrition status assessment  2) Basic complication management  (a) Basic emergency care, including basic child resuscitation  (b) Sick infant/child with danger signs  (c) Jaundice  (d) Feeding problem/low weight for age  (e) Diarrhea/dehydration  (f) Pneumonia  (g) Fever, including measles and dengue  (h) Ear problem and sore throat  (i) Anemia  (j) Severe acute malnutrition (SAM) without complication  3) Health education on danger signs/nutrition/immunization/child development | No |  |  |  |  |  |  |
| 39 | Outreach and Community based child curative care 1) Diarrhea management (ORS and Zinc) 2) Pneumonia management (antibiotics) 3) Detect danger signs for severe child illness (e.g. severe acute malnutrition) and refer 4) Screening of nutrition status with MUAC for children under 5 years of age | No |  |  |  |  |  |  |
|  | Total  (Among all the 39 interventions) | 8(Yes)/ 39(No)  (20·5 %) |  |  |  |  |  |  |

**Definition of color coding**

| Supported | All care tasks in a concerned practice category are supported by the Lao PDR Scope of Midwifery Practice |
| --- | --- |
| Partially supported | Not all care tasks in a concerned practice category are supported by the Lao PDR Scope of Midwifery Practice |
| No supported | No care tasks in a concerned practice category are supported by the Lao PDR Scope of Midwifery Practice |
| Undefinable | Care tasks of a concerned practice category have not been defined by existing standards and guidelines |

*1 Practice categories fully supported across all the service areas (history taking, physical exam, interpret test, education, counselling, and advice for uncomplicated cases, and education, counselling, and advice for cases with complications) are omitted from the table.

*2 International scope of midwifery practice does not require midwives to conduct sterilization procedures.

*3 International scope of midwifery practice requires midwives to conduct STI treatment in pregnancy.

*4 International scope of midwifery practice requires midwives to conduct screening (counselling and testing) for women in reproductive age.

*5 International scope of midwifery practice requires midwives to perform manual vacuum aspiration of the uterus up to 12 completed

weeks of pregnancy.

*6 International scope of midwifery practice requires midwives to conduct administration of antiretroviral drugs for reducing mother to child transmission, and antiretroviral therapy for treatment of HIV infection in pregnant women.

*7 International scope of midwifery practice requires midwives to conduct screening of diabetes.

*8 International scope of midwifery practice requires midwives to stabilize women in obstetric emergency and refer for treatment.
