# Supplementary material for: A regulatory gap analysis of midwifery to deliver essential reproductive, maternal, newborn, child and adolescent health services in Lao People’s Democratic Republic
Source: Lancet Reg Health West Pac. 2023 Dec 5;43:100960. doi: 10.1016/j.lanwpc.2023.100960 (PMC10749282; doi:10.1016/j.lanwpc.2023.100960)
Supplement: Translated abstract [file mmc2.pdf]

ບົດຄັດຫຍໍ້

ປະຫວັດຄວາມເປັນມາ

ໃນສາທາລະນະລັດ ປະຊາທິປະໄຕ ປະຊາຊົນລາວຜະດຸງຄັນແມ່ນຜູ້ໃຫ້ບໍລິການຫຼັກສໍາລັບການດູແລສຸຂະພາບຈະເລີນພັນ, ແມ່, ເດັກເກີດໃໝ່, ເດັກນ້ອຍ ແລະ ໄວໜຸ່ມ (RMNCAH). ພວກເຮົາໄດ້ວິເຄາະຂອບເຂດການປະຕິບັດງານຕາມລະບຽບຫຼັກການທີ່ອະນຸຍາດໃຫ້ຜະດຸງຄັນສາມາດໃຫ້ບໍລິການ RMNCAH ທີ່ຈໍາເປັນຕາມກໍານົດໄວ້ໃນລະດັບຊາດ.

ວິທີການ

ໄດ້ມີການປຶກສາຫາລືກັບພາກສ່ວນທີ່ກ່ຽວຂ້ອງ ແລະ ການທົບທວນເອກະສານໄດ້ຖືກດໍາເນີນຂຶ້ນເພື່ອກໍານົດໄດ້ຊ່ອງຫວ່າງການປະຕິບັດທີ່ຈໍາເປັນສໍາລັບ RMNCAH ແລະ ໜ້າວຽກງານການດູແລທີ່ຄາດວ່າຜະດຸງຄັນສາມາດປະຕິບັດໄດ້ໂດຍທີ່ບໍ່ຈໍາເປັນຕ້ອງມີທ່ານໝໍ. ເຊິ່ງໜ້າວຽກເຫຼົ່ານີ້ໄດ້ຖືກກໍານົດໄວ້ໃນ 1) ຊຸດບໍລິການສຸຂະພາບທີ່ຈໍາເປັນ (EHSP) ແລະ 2) 18 ມາດຕະຖານ ແລະ ຄໍາແນະນໍາແຫ່ງຊາດ. ຈາກນັ້ນ ພວກເຮົາຈຶ່ງໄດ້ສ້າງແຜນທີ່ເບິ່ງວ່າກົດລະບຽບຂອງຜະດຸງຄັນເຊິ່ງຂຽນໄວ້ໃນກອບແຜນວຽກດ້ານກົດໝາຍ ສໍາລັບການໃຫ້ບໍລິການທາງດ້ານວິຊາການໄດ້ສະໜັບສະໜູນການດູແລຕາມມາດຕະຖານທີ່ວາງອອກ ຫຼື ບໍ່ ເພື່ອກໍານົດໄດ້ຊ່ອງຫວ່າງດ້ານລະບຽບການ. ຂໍ້ມູນນີໄດ້ຖືກນໍາໃຊ້ເຂົ້າໃນການປັບປຸງບັນດາຂໍ້ກໍານົດ-ລະບຽບການຕ່າງໆ.

ຜົນໄດ້ຮັບ

ຕາມຂໍ້ກໍານົດ ແລະ ລະບຽບການ ຜະດຸງຄັນສາມາດໃຫ້ບໍລິການ 39 ລາຍການທີ່ນອນຢູ່ໃນການບໍລິການ RMNCAH ໂດຍບໍ່ມີທ່ານໝໍ ເຊິ່ງກວມເອົາ 1,100 ໜ້າວຽກ. ຈາກຂໍ້ມູນພື້ນຖານໃນການປະຕິບັດຕົວຈິງເຫັນວ່າ ມີພຽງ 8 ໃນ 39 ການບໍລິການ (20,5%) ແລະ 705 ໃນ 1,100 ຂອງໜ້າວຽກງານ (64.1%) ທີ່ຜະດຸງຄັນສາມາດໃຫ້ບໍລິການໄດ້ . 31 ລາຍການການສະໜອງການບໍລິການອື່ນແມ່ນບໍ່ອະນຸຍາດໃຫ້ຜະດຸງຄັນໃຫ້ບໍລິການ, ໃນນັ້ນ 83.9% (26) ຂອງໜ້າວຽກແມ່ນກ່ຽວຂ້ອງກັບການຂຽນໃບຢາ ແລະ ວາງຢາປິ່ນປົວ, 51.6% (16) ແມ່ນຕິດພັນກັບການສັ່ງກວດ ແລະ ການບົ່ງມະຕິ, 38.7% (12) ແມ່ນການບົ່ງມະຕິພະຍາດຕາມອາການ ແລະ 22,6% (7) ແມ່ນກ່ຽວຂ້ອງກັບບໍລິການທີ່ບໍ່ຕິດພັນກັບການໃຫ້ຢາ. ກະຊວງສາທາລະນະສຸກໄດ້ເຮັດວຽກຮ່ວມກັບຫຼາຍພາກສ່ວນທີ່ກ່ຽວຂ້ອງເພື່ອປັບປຸງລະບຽບການ ການຈັດປະຕິບັດວຽກຂອງຜະດຸງຄັນ, ການປັບປຸງນີ້ ເຮັດໃຫ້ມີການເພີ່ມຂຶ້ນຂອງລາຍການການບໍລິການ ແລະ ໜ້າວຽກຕາມລະບຽບການທີ່ກໍານົດ ມາເປັນ 35 ການບໍລິການ (94.9%) ແລະ 1,081 ໜ້າວຽກ (98.3%) ຕາມລໍາດັບ.

ຄຳອະທິບາຍຜົນໄດ້ຮັບ

ວິທີການໃໝ່ນີ້ໄດ້ຊ່ວຍກຳນົດຊ່ອງຫວ່າງຂອບເຂດການປະຕິບັດວຽກຂອງຜະດຸງຄັນຢ່າງເປັນລະບົບ ພ້ອມກັບຊ່ວຍປັບປຸງຂອບເຂດການປະຕິບັດວຽກໂດຍໃຊ້ຂໍ້ມູນ. ດັ່ງນັ້ນ, ການຄົ້ນຄວ້ານີ້ໄດ້ຊ່ວຍປັບປຸງລະບຽບຂອບເຂດການປະຕິບັດເພື່ອສະໜັບໜູນການບໍລິການສຸຂະພາບຈະເລີນພັນ, ແມ່, ເດັກເກີດໃໝ່, ເດັກເກີດໃໝ່, ເດັກນ້ອຍ ແລະ ໄວໜຸ່ມ ໃນຂັ້ນພື້ນຖານ. ວິທີການນີ້ສາມາດດັດປັບເພື່ອນຳໄປໃຊ້ກັບຂົງບໍລິການສຸຂະພາບອື່ນໆ , ຂົງເຂດການບໍລິການ ແລະ ປະເທດອື່ນໆ.

This translation in Lao was submitted by the authors and we reproduce it as supplied. It has not been peer reviewed. Our editorial processes have only been applied to the original abstract in English, which should serve as reference for this manuscript.
